# Congenital Chest Wall Deformities in Children: A Narrative Review

**DOI:** 10.3390/children13091265

**Published:** 2026-09-17

**Authors:** Małgorzata Kowalska, Hanna Grabowska, Michał Szostawicki, Michał Puliński, Tomasz Janowicz, Adam Hermanowicz

**Affiliations:** 1 Department of Pediatric Surgery and Urology, Medical University of Bialystok, Waszyngtona 17, 15-274 Bialystok, Poland; 2 Department of Surgery, Clinical Department of Pediatric Surgery and Urology, School of Public Health, University of Warmia and Mazury in Olsztyn Collegium Medicum, Żołnierska 18A, 10-561 Olsztyn, Poland

**Keywords:** chest wall deformity, pectus excavatum, pectus carinatum, Poland syndrome, vacuum bell, dynamic compression bracing, Nuss procedure, radiation-free imaging, quality of life, children

## Abstract

**Highlights:**

**What are the main findings?**

- Chest wall deformities in children span a wide spectrum—from pectus excavatum and carinatum (affecting ~1% of children) to rare, life-threatening anomalies—and are best understood as a genetically heterogeneous, mostly multifactorial group with clinically important syndromic associations rather than isolated cosmetic conditions.
- The most reproducible benefit of treatment lies in improved body image, self-esteem and quality of life, which are largely independent of anatomical severity, whereas a generalizable cardiopulmonary benefit of surgical correction remains unproven.

**What are the implications of the main findings?**

- Conservative remodelling (vacuum bell and dynamic compression bracing) should be first-line in suitable children, with minimally invasive, hybrid and modified-Ravitch surgery reserved for complex morphology.
- Surgical indications should integrate psychosocial burden alongside anatomical criteria, and early-onset deformity should prompt genetic referral and cardiovascular surveillance for associated connective-tissue disorders.

**Abstract:**

Congenital chest wall deformities encompass a broad spectrum of anomalies, from the common pectus excavatum and pectus carinatum to rare, life-threatening conditions such as sternal clefts, ectopia cordis, pentalogy of Cantrell, and asphyxiating thoracic dystrophy (Jeune syndrome). This narrative review synthesizes current evidence on their epidemiology and pathogenesis, genetics and syndromic associations, diagnostic assessment, and the full range of conservative and surgical management, with particular attention to developments of the past decade. Pectus deformities affect roughly 1% of children, and although familial clustering supports a genetic contribution, no single causative gene has been established; syndromic associations, especially connective-tissue disorders, remain clinically actionable and warrant cardiovascular surveillance. Conservative treatment has become first-line for suitable patients: the vacuum bell for pectus excavatum and dynamic compression bracing for pectus carinatum both achieve good results when compliance is maintained. Minimally invasive repair remains the surgical standard for pectus excavatum, complemented by the modified Ravitch and hybrid procedures for complex morphology, while emerging innovations and enhanced-recovery protocols continue to improve safety and recovery. The rarer midline and chondrodysplastic deformities demand individualized, often emergency, multidisciplinary care. Across the spectrum, the most reproducible benefit of treatment lies in body image, self-esteem and quality of life, whereas a generalizable cardiopulmonary benefit remains unproven. Management should be individualized to the deformity, the child’s physiology, and the psychosocial burden of disease.

## 1. Introduction

**Figure 1:**
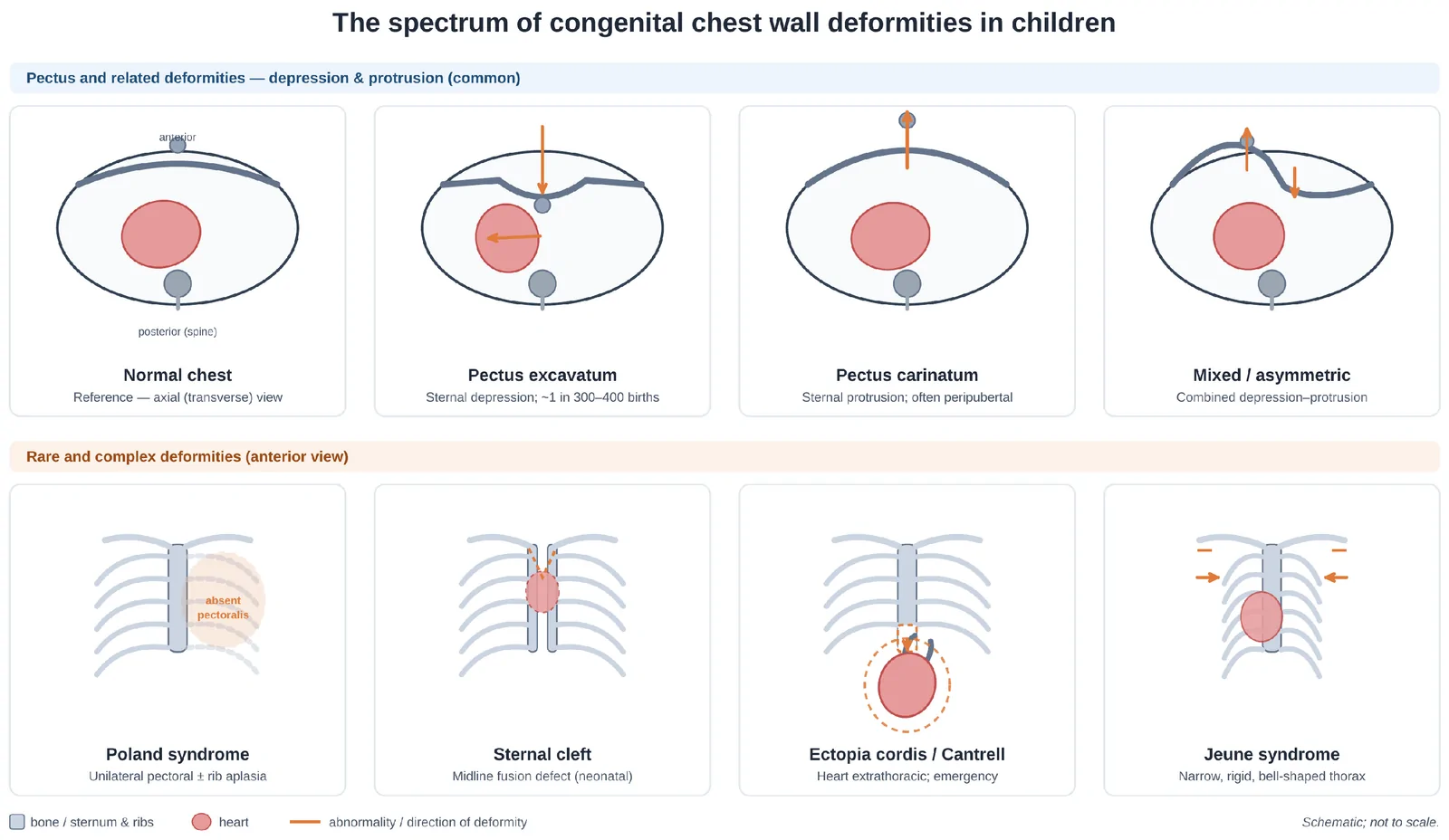
Schematic overview of the spectrum of congenital chest wall deformities in children. Top row (axial/transverse view): the common pectus and related deformities, showing the normal sternovertebral relationship, posterior sternal depression in pectus excavatum (with leftward cardiac displacement), anterior protrusion in pectus carinatum, and a combined asymmetric pattern. Bottom row (anterior view): the rarer and more complex deformities—unilateral pectoral ± rib aplasia in Poland syndrome, a midline sternal fusion defect, extrathoracic heart in ectopia cordis/pentalogy of Cantrell, and the narrow, rigid, bell-shaped thorax of Jeune syndrome.

Chest wall deformities in children represent a group of structural abnormalities that affect the shape and function of the thoracic cage. The prevalence of chest wall deformities varies globally, with pectus excavatum occurring in approximately 1 in 300–400 live births and showing a higher incidence in males. Epidemiological studies indicate that these conditions often manifest during early childhood or adolescence and are frequently associated with genetic and connective tissue disorders. Early recognition and appropriate management are essential because of the potential impact on cardiopulmonary function and psychosocial well-being. This review aims to provide an up-to-date, clinically oriented synthesis of congenital chest wall deformities in children, spanning the full spectrum of these conditions rather than a single entity or operative technique. Chest wall deformities are broadly classified into depression (pectus excavatum), protrusion (pectus carinatum), and mixed forms, alongside rarer entities such as Poland syndrome, sternal clefts, ectopia cordis, and Jeune syndrome (Figure 1).

## 2. Methodology

This study was conducted as a narrative review. A literature search was performed in PubMed up to 1 July 2026, using combinations of the terms: (“pectus excavatum” OR “pectus carinatum” OR “chest wall deformity” OR “Poland syndrome” OR “sternal cleft” OR “ectopia cordis” OR “Jeune syndrome” OR “thoracic insufficiency syndrome”) AND (child* OR pediatric OR paediatric OR adolescent*). The literature search was conducted exclusively in the PubMed/MEDLINE database; no other bibliographic databases were searched. The reference lists of the retrieved articles were hand-searched to identify additional sources.

Articles were eligible for inclusion if they were published in English and reported on human pediatric populations. Priority was given to publications from the last 10 years to reflect the current state of evidence. Landmark historical papers were retained irrespective of publication date where they established a diagnostic standard or an operative technique that remains in use or where they represent the primary description of a condition; these are cited to provide the developmental context of contemporary practice. Case reports were excluded, except for rare deformities for which higher-level evidence was not available. Conference abstracts and non-peer-reviewed sources were excluded from the study. Given the narrative design, no formal risk-of-bias assessment or PRISMA-based selection process was applied, and the synthesis is qualitative.

## 3. Epidemiology

Pectus deformities (PDs) are the most common congenital anomalies of the anterior chest wall, affecting approximately 1% of the population [1]. This estimate is supported by the first systematic review and meta-analysis on the subject, published by Pu et al., which pooled data from 34 studies identified from an initial 1510 records [2]. In that analysis, the pooled prevalence was 0.5% (95% CI: 0.3–0.9%) for pectus excavatum (PE) and 0.6% (95% CI: 0.5–0.8%) for pectus carinatum (PC), with an overall prevalence of PDs of 1.2% (95% CI: 0.9–1.5%). Rib anomalies were considerably rare, with a pooled prevalence of 0.05% (95% CI: 0.01–0.16%).

Both deformities show a marked male predominance. For PE, males are reported to be affected three to five times more often than females, and incidence rates of up to 8 per 1000 live births have been reported in the literature [1]. The incidence of PC is estimated at approximately 1 per 2500 live births, with a four-fold male preponderance [1]. It should be noted that these figures represent different epidemiological measures: the pooled prevalence of ~0.6% derives mainly from screening of school-age children and adolescents, whereas the incidence of approximately 1 in 2500 refers to detection at birth. Because pectus carinatum characteristically manifests during the pubertal growth spurt, its prevalence among older children exceeds its incidence recorded at birth, which reconciles the apparent discrepancy.

Poland syndrome is a rare entity, with reported incidence estimates ranging from 1 in 17,000 to 1 in 100,000 births; the true incidence is likely underestimated, as the condition is inconsistently recorded and frequently escapes diagnosis [3,4,5]. Males are affected approximately three times more often than females, and the right side is affected twice as often as the left side [4]. Sternal clefts, ectopia cordis, and pentalogy of Cantrell are considerably rarer and present as neonatal emergencies requiring early surgical intervention [6].

## 4. Genetics and Associated Syndromes

Familial clustering most consistently supports the genetic contribution to pectus deformities. A positive family history of chest wall abnormality is reported in approximately 35–45% of patients with pectus excavatum (PE), and segregation analysis within affected families indicates that no single Mendelian model accounts for the observed disease transmission. Autosomal dominant, autosomal recessive, and X-linked patterns have all been proposed, with variable expressivity and incomplete penetrance [7,8]. Therefore, PE is best regarded as a genetically heterogeneous, most likely multifactorial trait rather than a single-gene disorder.

Attempts to identify causative genes have thus far produced candidate loci rather than established ones. The most recent systematic review identified 14 distinct genetic loci reported across heterogeneous study designs (family-based exome sequencing, case reports, candidate-gene analyses, murine models, and linkage analysis implicating chromosome 18q) and grouped them into seven biological pathways relevant to chest wall morphogenesis [9]:

- Extracellular matrix and collagen metabolism (*COL5A1*, *COL1A1*, and *COL27A1*): *COL5A1* regulates collagen fibril diameter, which is critical for cartilage ultrastructure; *COL1A1* encodes the principal structural collagen of bone and costal cartilage; *COL27A1* is cartilage-specific and essential for its structural integrity. Altered collagen composition may change the mechanical properties of the chest wall and permit abnormal growth of the chest wall.
- TGF-β/BMP signalling (*SMAD4* and *TGFB3*): *SMAD4* is the central intracellular mediator of both cascades, and *TGFB3* is a key upstream ligand; together, they regulate chondrocyte proliferation, differentiation, and hypertrophy during endochondral ossification.
- Cartilage development and homeostasis (*ACAN*, *GPR126*, and *GAL3ST4*): Aggrecan confers compressive resistance to cartilage, *GPR126* regulates chondrocyte maturation, and *GAL3ST4* modifies the proteoglycans.
- RAS/MAPK growth-plate signalling (*SOS1*, *PTPN11*, and *NF1*): Governs chondrocyte proliferation; hyperactivation in Noonan-spectrum disorders produces abnormal rib growth and increased chest wall laxity.
- Skeletal patterning (*TGDS*, *COL27A1*, and *COL1A1*): Disrupted thoracic patterning or altered ossification timing may increase susceptibility.
- Transcriptional regulation (*REST*): A master repressor of neuronal genes in non-neuronal tissues, implicated in mesenchymal stem cell differentiation, although its mechanism in PE remains unclear.
- Neuromuscular support (*BICD2*): Reduced anterior chest wall support may aggravate pre-existing structural vulnerability.

Notably, these pathways are not mutually exclusive; *COL1A1* and *COL27A1* are assigned to both collagen metabolism and skeletal patterning, and their convergence on chondrocyte biology and extracellular matrix integrity is the most consistent theme to emerge from the genetic literature to date. Critically, no population-level genome-wide association study of isolated PE has been performed, and none of the included studies reported allele frequencies, effect sizes, or odds ratios [9].

The complexity of this picture is illustrated by the largest family-based sequencing study to date, in which germline exome sequencing was performed in ten families, each containing three affected members (30 familial PE cases) [10]. Candidate variants in *REST*, *SMAD4*, and *COL5A* segregated with the phenotype within individual pedigrees, but no variant was shared between families. The authors concluded that familial PE is unlikely to be explained by a shared single variant in known genes, recommending polygenic, non-coding, and epigenetic analyses as the next steps [10]. This finding tempers the interpretation of earlier candidate-gene reports and argues against attributing PE to any individual gene in the current clinical practice.

In contrast, syndromic associations are well established and clinically actionable. A systematic review identified 20 distinct congenital genetic disorders associated with PE, grouped into chromosomal disorders (n = 3), connective tissue diseases (n = 5), neurological disorders (n = 3), syndromic disorders (n = 8), and other conditions (n = 1) [11]. In a subset of these—including Marfan syndrome, Loeys–Dietz syndrome, Aarskog–Scott syndrome, Shprintzen–Goldberg syndrome, Malan syndrome and *ABCA3*-related disease—PE occurs in more than 40% of affected individuals, making the deformity a clinically useful phenotypic marker [11]. Recognizing that the referral of patients with PE to clinical genetics is frequently delayed, the same authors proposed a practical scoring list to guide such referrals [11]. Overlapping gene groups have long been described in the syndromes most familiar to the operating surgeon: *FBN1* (Marfan), *TGFBR1*/*TGFBR2* (Loeys–Dietz), *FBN2* (congenital contractural arachnodactyly), *TBX5* (Holt–Oram), and *PTPN11*, *KRAS*, *SOS1*, and *RAF1* (Noonan) [8].

The cardiovascular implications of these associations are the principal reason why genetic assessment is not merely academic. In the pooled meta-analysis by Pu et al., the incidence of Marfan syndrome among patients with pectus deformities was 11.4% (95% CI: 3.5–31.4%), mitral valve prolapse 19.7% (95% CI: 10.2–34.7%) and aortic root dilatation 12.6% (95% CI: 6.8–22.2%) [2]. In a single-center series of 1215 operated patients, 2.8% had a genetic diagnosis of Marfan syndrome, and a further 17% displayed clinical features suggestive of it [12]. Given that aortic pathology is the principal determinant of morbidity and mortality in Marfan syndrome, echocardiographic assessment of the aortic root and mitral valve should be considered in patients presenting with pectus deformities, particularly before surgical correction.

Age at presentation is emerging as a practical triage criterion for this purpose. Only about one—fifth of PE cases become apparent within the first decade of life and are thus of truly congenital origin; in a prospective screening study in which children under 11 years of age presenting with PE were independently assessed by two clinical geneticists, pathogenic genetic variations were identified in 8 of 18 patients (44%)—three syndromic disorders (Catel–Manzke syndrome and two cases of Noonan syndrome), three chromosomal disorders (16p13.11 microduplication, 22q11.21 microduplication, and a gain at 1q44), one connective tissue disease (Loeys–Dietz syndrome) and one neuromuscular disorder (a pathogenic *BICD2* variant) [13]. Although the sample was small, the yield is high enough to support the authors’ recommendation that early-onset PE, in contrast to deformity that becomes apparent during puberty, should prompt referral for genetic counselling [13].

In summary, while the molecular architecture of isolated pectus deformity remains undefined, the clinician’s practical obligations are clear: to take a family history, recognize the syndromic phenotypes in which PE is over-represented, arrange cardiovascular imaging where a connective tissue disorder is plausible, and refer early-onset cases for genetic evaluation. Resolving the underlying genetic architecture will require large-scale international GWAS, which currently represents the single most conspicuous gap in this field [9].

## 5. Diagnostics and Imaging

Preoperative assessment of pectus deformities has evolved from simple clinical inspection and descriptive caliper measurement of the chest to quantitative, image-based documentation [14]. Before surgical correction, it is useful to establish not only the depth of the depression but also the presence of asymmetry, costal flaring, and sternal torsion, together with any cardiac compression or displacement [14]. No single modality satisfies all of these requirements, and the choice of imaging in children is additionally constrained by the need to limit ionizing radiation.

The Haller index is the most widely used measure of severity. First described in 1987, it is calculated by dividing the maximum transverse diameter of the chest at the deepest point of the deformity by the minimum anteroposterior distance. Notably, the deepest point of depression is not necessarily at the sternum and may lie near the interchondral joints [1,15]. A value of ≥3.25 has conventionally qualified a patient for operative correction [1]. However, its limitations are now well recognized: the index bears no direct relationship to physical or psychological complaints, and it varies with thoracic shape, the vertebral level at which it is measured, and age and sex [1].

These shortcomings prompted the development of the Pectus Correction Index by St Peter et al. in 2011 [16]. Thoracic width was deliberately omitted from this calculation because it correlates only weakly with the depth of the deformity. A horizontal line is drawn across the anterior spine, and on the same slice, two distances are measured: the minimum distance from the posterior sternum to the anterior spine and the distance from the inner margin of the anterior chest to that line. The difference, divided by the maximum prominence of the chest and multiplied by 100, yields the index [1]. A correction index of 10% has been proposed to define the deformity, while a value of approximately 28% corresponds to a Haller index of 3.25, thus representing an equivalent operative threshold [1]. Because neither index accounts for cardiopulmonary compression or morphological asymmetry, the cardiac compression index and sternal depression index were subsequently introduced in an attempt to incorporate cardiac involvement into the classification [1]. However, no anatomical index can capture subjective symptoms or physiological consequences of altered cardiac geometry. In most countries, the decision to operate now rests on symptomatology and demonstrable cardiopulmonary compression rather than anatomical thresholds alone [1]. The available indices are listed in Table 1.

Computed tomography remains the reference modality, permitting the calculation of the pectus indices, three-dimensional reconstruction of the thoracic wall, and identification of cardiac compression, chest asymmetry, and sternal torsion [14]. Its principal drawback in the pediatric population is the radiation dose, which is typically 1–2 mSv for a full chest study. A very low-dose protocol restricted to five to seven slices centered on the point of maximal sternal depression reduces this to approximately 0.35 mSv while still allowing the Haller index to be calculated and the local anatomy to be displayed [14]. Chest radiography offers a further reduction in dose and cost and supports several indices, including the Welch index, vertebral index, body–manubrium–xyphoid index, and Haller and correction indices. However, it is unsuitable for the assessment of asymmetry or sternal torsion [14]. Magnetic resonance imaging is radiation-free, permits the calculation of the same indices, and uniquely allows the assessment of cardiac function, with real-time and exercise protocols providing dynamic information. However, its availability and cost remain limited [14]. Echocardiography identifies structural cardiac changes but has limited accuracy in the functional evaluation of these patients, and medical photography combined with caliper measurement remains a useful adjunct for qualitative and quantitative documentation [14].

Three-dimensional optical surface imaging is the most significant recent development and is of particular relevance to pediatric practice. Most systems use structured light projection, in which distortion of a projected light pattern by the thoracic geometry allows protrusions and depressions to be measured; acquisition takes seconds and involves no ionizing radiation [14]. Indices derived from the skin surface correlate with their conventional counterparts, and equivalent operative thresholds have been established: an external Haller index of ≥1.83 corresponds to a conventional Haller index of ≥3.25, and an external correction index of ≥15.2% to a conventional correction index of ≥28.0% [14]. Optical imaging can additionally quantify other morphological features and predict the presence of cardiac compression without recourse to CT, and when combined with predictive algorithms, it may assist both in operative decision-making and in visualizing the expected aesthetic result [14]. Its accuracy remains limited compared to that of cross-sectional imaging, and it does not display internal structures.

Automation is also advancing in parallel. Deep-learning approaches have been applied to the automatic calculation of pectus indices, including a UNet++ architecture evaluated on a database of 269 CT scans that computed the Haller index, correction index, and asymmetry index without manual input [14]. A separate group proposed a set of four geometric measures–eccentricity, flatness, circularity, and rotation indices–that quantify both depression and asymmetry through a fully automatic segmentation pipeline [14]. Such tools promise to reduce inter-observer variability, although none have yet entered routine practice.

Finally, it should be emphasized that this quantitative apparatus has been developed almost entirely for pectus excavatum. For pectus carinatum, the Haller index has been adapted by substituting the maximum anteroposterior length of the chest wall for the minimum sternovertebral distance [1]; however, no validated severity index with an established operative threshold exists, and assessment remains largely clinical, a disparity that mirrors the wider imbalance of evidence between the two deformities.

## 6. Psychosocial Aspects and Cardiorespiratory Function

### 6.1. Psychosocial Burden

Although pectus deformities are frequently dismissed as cosmetic, their psychosocial impact is now recognized as a central component of the disease, rather than a secondary concern. The progression of both pectus excavatum and pectus carinatum coincides with adolescence, a critical period for identity and self-esteem development. Across multiple cohorts, adolescents and young adults with chest wall deformities consistently report impaired body image, reduced self-esteem, social avoidance, and diminished quality of life, even in the absence of a diagnosable psychiatric disorder [24]. The landmark multicenter study by Kelly et al. established that surgical repair markedly improves body image and perceived capacity for physical activity, and this benefit has been reproduced with disease-specific instruments, such as the Pectus Excavatum Evaluation Questionnaire and the Nuss Questionnaire modified for adults [24,25].

A key recent observation is that the magnitude of this burden is largely independent of the anatomical severity. In a pediatric cohort of 102 evaluated patients (57 surveyed; median age, 14 years; 86% male), impaired quality of life, anxiety, and depressive symptoms showed no significant relationship with the Haller index [26]. The authors argue that reliance on anatomical and physiological thresholds for operative candidacy contributes to inequitable access to care and that consensus guidance integrating anatomical severity, symptoms, psychosocial distress, and progression would be preferable [26]. This position—that the psychosocial dimension may warrant broadening the criteria for intervention—is among the more consequential debates currently shaping practice.

### 6.2. Cardiorespiratory Function

The physiological case for correction is less settled than the psychosocial one. Severe pectus excavatum can compress the right heart and is associated with structural and functional cardiac changes, mitral valve prolapse, conduction abnormalities, and arrhythmias. In the pooled meta-analysis by Pu et al., the incidence of mitral valve prolapse, abnormal electrocardiogram, aortic root dilatation, and cardiac anomalies among patients with pectus deformities was 19.7%, 13.4%, 12.6%, and 11.1%, respectively [2]. A single-center series of four patients with pre-existing cardiac disease (pericarditis, mitral valve prolapse, ventricular fibrillation arrest, and second-degree atrioventricular block) illustrated that in selected cases, severe sternal depression can aggravate underlying cardiac pathology, and that operative correction may help resolve it [27].

However, whether this correction improves cardiorespiratory fitness in the general pectus population is not supported by the best available synthesis. In a systematic review and meta-analysis of 15 non-randomized studies (1598 screened), Media et al. found no statistically or clinically significant improvement in maximal or peak oxygen uptake ($\dot{V}O_{2}\max/\dot{V}O_{2}\mathrm{peak}$) following surgical correction and could not demonstrate any consistent physiological benefit [28]. This finding contradicts the long-standing physiological rationale for surgery and individual reports of improved exercise capacity; the authors note that the evidence base is limited to non-randomized studies of variable quality. Taken together with the psychosocial data, it suggests that the principal, reproducible benefit of correction lies in body image, self-esteem, and quality of life, while a generalizable cardiopulmonary benefit remains unproven and is probably confined to a subset of patients with demonstrable cardiac compression.

## 7. Conservative Treatment

Nonoperative management has moved from a marginal role to a first-line option in appropriately selected children, driven by the recognition that a flexible, growing chest wall can be remodelled by sustained external force. The two principal modalities are mirror images of one another: the vacuum bell pulls the depressed sternum of pectus excavatum outward, whereas dynamic compression bracing presses the protruding sternum of pectus carinatum inward.

### 7.1. Vacuum Bell Therapy for Pectus Excavatum

The vacuum bell, introduced by Schier, Bahr, and Klobe, applies negative pressure over the anterior chest to progressively lift the sternum [29]. Two decades of clinical use have established it as a safe and potentially definitive alternative to surgery in carefully selected patients. In an international survey, 72% of the Chest Wall International Group members across 47 institutions reported using the device, although validated guidelines are still lacking [30]. The efficacy of these treatments depends heavily on patient selection and adherence. In the largest long-term series to date, 259 children followed for a median of 64 months, 63.7% completed treatment, of whom 52.1% achieved a successful correction; complications occurred in 22.8% but were minor, and recurrence after successful treatment was rare (2.3%) [31]. Greater daily wear, longer total duration, and overnight use predicted success, whereas deeper depression, highly flexible chest wall, and symptomatic deformity predicted poorer outcomes [31]. Two findings are of particular practical relevance: among children treated while awaiting a Nuss procedure, 26.7% ultimately no longer required surgery, supporting the use of the vacuum bell as a bridging or even substitutive therapy, and breast development led 39.3% of female patients to abandon treatment, underscoring the importance of early initiation [31]. The therapy appears to be most effective in younger children; under the age of 10, it may reasonably represent the first step of specific treatment [30].

### 7.2. Dynamic Compression Bracing for Pectus Carinatum

Because pectus carinatum is more amenable to external molding than pectus excavatum, bracing has become the treatment of choice for flexible protrusion deformities. The largest comparative experience, from a single high-volume center treating 738 patients over a decade, reported that dynamic compression bracing achieved successful correction in 73.8% of the 553 patients who completed treatment, with treatment failure in 13.6% [32]. Success fell as the pressure required for initial correction rose (from 84.2% to 67.3%), and Marfan and Poland syndromes were associated with unfavorable outcomes [32]. In the same cohort, open Ravitch repair produced a higher success rate (92.4%) but at a substantially greater cost in morbidity (complications in 32.4% of patients, reoperation for complications in 6.7%, and relapse in 7.6%), leading the authors to recommend bracing as first-line therapy and to reserve surgery for brace failure or rigid deformity [32]. Beyond correcting the contour, bracing improves quality of life, self-esteem, and physical symptoms, with most of the benefit accruing within the first 6–12 months [33]. Across every series, the recurring limitation is the same: outcomes depend on compliance, and motivation is as important a determinant of success as any anatomical variable [32].

### 7.3. Physiotherapy and Adjunctive Measures

Posture-correcting exercises and breathing training are commonly recommended as adjuncts intended to improve posture and thoracic mobility rather than to correct bony deformity and are best regarded as a complement to bracing or the vacuum bell rather than a stand-alone treatment [34].

## 8. Surgical Treatment and Techniques

Minimally invasive repair of pectus excavatum (MIRPE), commonly referred to as the Nuss procedure, has become the preferred operative approach for most children and adolescents requiring surgical correction of pectus excavatum. The technique achieves anterior displacement and progressive remodelling of the sternum without extensive costal cartilage resection or formal sternal osteotomy [35]. However, its contemporary form differs substantially from the original procedure introduced in 1998. Thoracoscopic guidance, individually modelled bars, multiple bar configurations, improved bar stabilization, safer mediastinal passage, and morphology-adapted entry points have progressively increased the reproducibility of MIRPE. A detailed discussion of the selected technical modifications and their practical application in pediatric patients was recently presented by Szostawicki et al. [36]. Comparative studies and meta-analyses have generally demonstrated shorter operative times and lower intraoperative blood loss after MIRPE than after open repair, while differences in overall complication rates in pediatric populations are less consistent [37]. Thus, MIRPE should not be regarded as a single standardized maneuver but as an evolving platform that can be adapted to the depth, length, rigidity, and rotational component of the deformity.

Despite the predominance of MIRPE, the modified Ravitch procedure plays an important role in contemporary chest wall surgery. It should no longer be considered merely a historical predecessor of the Nuss technique, but rather a morphology-specific option for patients in whom isolated retrosternal bar placement may not provide adequate correction. Potential indications include markedly rigid deformities, severe sternal rotation, complex asymmetric pectus excavatum, mixed excavatum–carinatum abnormalities, pectus arcuatum, extensive calcification of the costal cartilages, previous cardiac or chest wall surgery, and recurrent deformity after failed MIRPE [38,39,40]. Analysis of the Society of Thoracic Surgeons database showed that open repair was associated with longer operating times and hospitalization; however, after adjustment for patient and procedural factors, the type of repair was not independently associated with 30-day complications [39]. A recent institutional review covering 2016–2024 similarly demonstrated that Ravitch repair is now used selectively, particularly in older patients, redo operations, and anatomically complex deformities, while remaining a safe and effective reconstructive option [40].

Hybrid operations combine limited open release of the deformed costal cartilages and, when necessary, corrective sternal osteotomy with retrosternal support using one or more Nuss bars. The rationale is to decrease the mechanical forces required to elevate a severely rotated or rigid sternum while preserving the internal stabilization and remodelling advantages of MIRPE. In a series of 80 patients with asymmetric pectus excavatum treated using a combined Ravitch–Nuss approach, Pawlak et al. reported stable and satisfactory correction in 88% of patients, although postoperative complications, predominantly transient pleural complications, were relatively frequent [41]. More recently, Masai et al. described successful initial correction in nine patients with extremely severe deformities using a combined procedure, with a reduction in the median Haller index from 15.4 to 3.29 [42]. These results support the feasibility of hybrid reconstruction; however, the evidence remains limited to retrospective series and highly selected patients. Therefore, hybrid repair should be performed in specialized centers after a detailed three-dimensional assessment of chest wall morphology rather than routinely.

One of the most relevant safety innovations in MIRPE is the temporary elevation of the sternum before mediastinal dissection and bar passage. Crane techniques using transosseous sutures or hooks, dedicated external retractors, subxiphoid lifting devices, and the vacuum bell can enlarge the retrosternal space, improve thoracoscopic visualization, and reduce the force required to pass the introducer [43,44]. Quantitative imaging studies have confirmed that crane elevation can produce a substantial temporary increase in the anteroposterior diameter of the chest [44]. This strategy is particularly valuable in deep, rigid, or asymmetric deformities and reoperations, in which adhesions may bring the pericardium or heart into direct proximity to the posterior surface of the sternum. Nevertheless, most supporting evidence remains observational, and sternal elevation should complement—not replace—direct thoracoscopic control and meticulous mediastinal dissection.

The Magnetic Mini-Mover Procedure represents a fundamentally different concept. It uses an implanted sternal magnet and an external magnetic brace to generate gradual anterior traction and remodelling of the chest wall [45]. Experimental development and a subsequent FDA-sponsored multicenter trial demonstrated the technical feasibility of this strategy, particularly in younger patients with a compliant chest wall [45,46]. However, correction develops slowly and depends on prolonged use of the external device, appropriate patient selection, and adherence. The absence of robust long-term comparative evidence means that magnetic correction remains investigational and cannot currently be considered an alternative to the established MIRPE.

Bioresorbable implants are another potential direction for development. Polylactide plates, struts, and absorbable bar-stabilization systems have been evaluated as a means of providing temporary support while avoiding permanent metallic materials or a separate procedure for implant removal [47,48]. Early pediatric series demonstrated acceptable short-term stability and cosmetic outcomes but involved a small number of patients. Moreover, absorbable stabilizers have shown greater susceptibility to deformation or breakage than conventional metallic fixations [49]. At present, bioresorbable materials may be considered for selected fixation components, but there is insufficient evidence to replace load-bearing metallic pectus bars. Future development will require materials that combine predictable degradation with adequate mechanical strength, low inflammatory potential, and compatibility with imaging.

Finally, contemporary innovations extend beyond operative hardware. Enhanced Recovery After Surgery protocols integrate preoperative education, standardized anesthesia, multimodal opioid-sparing analgesia, early mobilization, early oral intake, respiratory physiotherapy, and objective discharge criteria. Wharton et al. demonstrated that implementation of an ERAS pathway reduced mean hospital stay after the Nuss procedure from 3.49 to 2.90 days, decreased early postoperative pain, and reduced urinary catheter use without increasing emergency visits or readmissions [17]. Programs combining ERAS with regional anesthesia and intercostal nerve cryoablation have enabled next-day discharge in many patients [50]. Cryoanalgesia has become a major and rapidly developing component of perioperative pain management; given the breadth of the topic, its indications, technical details, and potential complications fall beyond the scope of this review and are best addressed in separate, dedicated papers.

Therefore, the future management of pectus excavatum is unlikely to be based on a single universal operation. Rather, it will depend on an individualized algorithm incorporating age, chest wall flexibility, asymmetry, sternal rotation, previous surgery, cardiopulmonary effects, and patient expectations. MIRPE will remain the principal minimally invasive platform, whereas modified Ravitch and hybrid procedures will continue to provide indispensable solutions for complex anatomical deformities. Sternal elevation, advanced imaging, bioresorbable materials, gradual remodelling technologies, and comprehensive ERAS pathways are expected to further improve procedural safety, recovery, and long-term patient-centered outcomes.

## 9. Rare and Complex Deformities

Beyond pectus deformities, pediatric surgeons encounter a group of far rarer anomalies whose management differs fundamentally, dominated by neonatal urgency, reconstruction, and, for chondrodysplasias, the preservation of thoracic growth rather than the correction of contour.

### 9.1. Poland Syndrome

Poland syndrome is a unilateral malformation combining absence or hypoplasia of the pectoralis major (typically its sternocostal head) with variable ipsilateral hand (symbrachydactyly) and chest wall involvement, including hypoplastic or absent ribs and breast [4]. It is rare, with reported incidence estimates ranging from 1 in 17,000 to 1 in 100,000 births, and is probably underdiagnosed. Males are affected approximately three times as often as females, and the right side is affected twice as often as the left side [3,4]. The leading pathogenic hypothesis is the subclavian artery supply disruption sequence, an interruption of blood supply to the affected side around the sixth week of gestation, the severity of which tracks the level of vascular occlusion [4]. Because the osseous thorax rarely produces functional respiratory compromise, surgery is undertaken predominantly for aesthetic and psychosocial reasons and demands an individualized, multidisciplinary reconstruction combining custom implants, autologous tissue, and, in females, breast reconstruction [4,5]. Increased oncological vigilance, particularly for breast cancer on the affected side, is advised [4].

### 9.2. Sternal Clefts

A sternal cleft results from the failure of ventral midline fusion of the sternal bars during early embryogenesis and may be complete or partial (with superior clefts being more common) [6]. In its isolated form, the heart is covered by skin, and the prognosis is favorable because primary approximation of the sternal bars is best tolerated in the neonatal period when the chest wall is most compliant, which is an argument for early repair [6].

### 9.3. Ectopia Cordis and Pentalogy of Cantrell

Ectopia cordis, the extrathoracic location of the heart, is exceptionally rare and is classified according to the position of the displaced heart as cervical, thoracic, thoracoabdominal, or abdominal [6]. It frequently occurs within the pentalogy of Cantrell, which is the association of a midline supraumbilical abdominal wall defect, lower sternal defect, anterior diaphragmatic defect, pericardial defect, and an intracardiac anomaly [6]. These present as neonatal emergencies requiring immediate multidisciplinary intervention, and the prognosis is guarded, being determined chiefly by the intracardiac malformation and the degree of cardiac exposure [6].

### 9.4. Jeune Syndrome (Asphyxiating Thoracic Dystrophy)

Jeune syndrome is an autosomal recessive skeletal ciliopathy (with mutations in intraflagellar-transport genes such as IFT80 and DYNC2H1) affecting roughly 1 in 100,000–130,000 live births, characterised by a narrow, rigid, bell-shaped thorax with short horizontal ribs [51]. The constricted chest cannot support normal lung growth or respiration, producing thoracic insufficiency syndrome, the inability of the thorax to support normal respiration or lung growth, a concept introduced by Campbell [52]. Management is not directed at cosmetic correction but at expanding the thoracic volume to permit lung growth. Expansion thoracoplasty with a vertical expandable prosthetic titanium rib (VEPTR), the only device approved for this indication, is repeatedly lengthened as the child grows [18,53]. Nevertheless, outcomes remain guarded, with substantial perioperative morbidity and mortality reflecting the underlying respiratory and often renal and hepatic involvement [53].

## 10. Conclusions

This review has several limitations. The literature search was restricted to a single database (PubMed/MEDLINE); relevant studies indexed only in other databases, such as Scopus, Web of Science, or the Cochrane Library, may therefore have been missed. In addition, as a narrative rather than a systematic review, no formal risk-of-bias assessment or PRISMA-based selection process was applied, and the synthesis is qualitative. These factors may introduce selection bias, and the findings should be interpreted with this in mind.

Taken together, this heterogeneous group underscores that “chest wall deformity” spans a spectrum ranging from the common and largely cosmetic to the rare and life-threatening, and that a single therapeutic philosophy cannot serve all. Where pectus excavatum and carinatum are addressed electively and increasingly by conservative means, Poland syndrome centers on individualized reconstruction, midline fusion defects on emergency neonatal surgery, and thoracic chondrodysplasias on the preservation of thoracic growth itself. What unites them is the principle that runs through this review: management must be individualized to the specific deformity, the child’s age and physiology, and the psychosocial as well as the anatomical burden of disease. The rarity of these conditions also means that the evidence base is correspondingly thin, largely comprising case series and single-center experiences, and that international registries and referral to specialist centers remain the most realistic route to improving outcomes for the affected children.

## Figures and Tables

**Table 1:** Indices used to quantify the severity and morphology of pectus deformities.

Index	Modality	Definition/Formula	Threshold/Reference Values	Comment
Haller index (HI)	CT; chest radiograph; MRI	HI = T/A. T = widest internal transverse diameter of the ribcage; A = shortest distance between the vertebra and the sternum.	>3.25 conventionally indicates operative candidacy	The gold standard, but the 3.25 cut-off has never been independently validated; alternative upper limits of 2.7, 3.1 and 3.2 have been proposed. Varies with age, sex, thoracic shape and respiratory phase; ignores asymmetry and cardiac compression. Radiograph-derived HI correlates closely with CT and reduces dose [1,14,15,17].
Correction index (CI/PCI)	CT; chest radiograph	A horizontal line is drawn across the anterior spine. On the same slice, the minimum distance from the posterior sternum to the anterior spine and the distance from the inner margin of the anterior chest to that line are measured; their difference is divided by the maximum prominence of the chest and multiplied by 100.	≥10% defines the deformity; ≈28% corresponds to a Haller index of 3.25	Independent of chest width. One of the few indices to have been independently validated [17,18].
Welch index	Chest radiograph	Depression ratio D1/D2; deformity grade (1-ratio) × 10, with additions for a rib angle > 25° or a cardiothoracic ratio > 50%.	≥5 recommended for operative repair	Graded 1–10 severity scale derived from a large historical series [19].
Vertebral index (lower/upper)	Chest radiograph	Ratio of the sternovertebral distance to the sagittal diameter of the vertebral body (lower VI at the xiphisternal, upper VI at the sternomanubrial junction).	Lower VI cut-off > 27%	Lower VI is age-dependent; upper VI is age-independent; both relate sternal approximation to the spine [19].
Body-manubrium (xyphoid) index	Chest radiograph	BM = ossified sternal-body length ÷ manubrium length; BMX additionally includes the xyphoid.	BM 2.16; BMX 2.73	Requires ossified sternal segments; a short sternal body may help predict the number of bars needed [19].
Frontosagittal index	Chest radiograph	(Minimum sagittal chest diameter ÷ maximum internal transverse diameter) × 100.	Preoperative cut-off < 29	Increases after correction; a plain-radiograph alternative to CT indices [19].
Titanic index (TI)	CT	Percentage of the length of the sternum lying behind the anterior costal line.	Mean 37%; >66.5% predicts the need for more than two bars (sensitivity 93%, specificity 92%)	Quantifies the cephalocaudal extent of the excavation rather than its severity at the deepest point. Correlates only weakly with HI and CI, so carries independent information. A tool for operative planning, not for establishing candidacy [20].
Sternal depression index (SDI)	CT	SDI = C/B. C = maximal internal sagittal diameter of the left hemithorax; B = minimal distance from the anterior surface of the vertebral column to the posterior border of the deepest portion of the sternum.	<2.4 mild; 2.4–2.9 moderate; >2.9 severe (mean 2.7 ± 1.4)	Correlates with the cardiac rotation angle (r = 0.75). Mean absolute sternal depression in the source series was 21 ± 7 mm [19].
Depression index (DI)	CT	Absolute depth of sternal depression divided by the transverse vertebral-body diameter (measured at T9–T11), the latter serving as a morphometric surrogate for patient size.	No fixed cut-off; scaled continuous measure	Independent of thoracic diameters; correlates with the Haller and correction indices and, in the original study, matched clinicians’ subjective severity ranking better than either [21].
Haje width-length index (WLI)	Coronal CT	WLI = W/L, where W = maximum width of the ossified sternal body and L = its length.	Mean 0.420 in controls; >0.446 in pectus excavatum	Higher values denote a wider sternal body, with possible implications for prognosis and choice of procedure [19].
Asymmetry index	CT	Asymmetry index = (R/L) × 100, where R and L are the anteroposterior distances between the anterior and posterior ribs on the right and left sides; best measured at the sternomanubrial junction.	100 denotes a symmetric chest; departure from 100 quantifies asymmetry	Combining the Haller index with the asymmetry index improves detection of asymmetric pectus excavatum; computed alongside the Haller and correction indices in automated deep-learning pipelines [19].
Cardiac compression index (CCI)	CT; MRI	CCI = transverse diameter of the heart ÷ minimum anteroposterior diameter of the heart (at the level of the xiphoid).	Higher values indicate greater cardiac compression; significant for diagnosis regardless of age	One of the cardiac deformity indices proposed by Kim et al. to bring cardiac involvement—which no purely skeletal index captures—into the assessment [22].
External Haller index	3D optical surface imaging; calipers	Analogue of the Haller index computed from the external skin-surface contour rather than internal bony landmarks.	≥1.83 corresponds to a conventional Haller index ≥ 3.25	Radiation-free and repeatable, well suited to follow-up during conservative treatment [14].
External correction index	3D optical surface imaging	Analogue of the correction index derived from the external skin-surface contour.	≥15.2% corresponds to a conventional correction index ≥ 28.0%	Radiation-free; may also predict the presence of cardiac compression without CT [14].
Modified Haller index (carinatum)	CT; chest radiograph	Haller index using the maximum anteroposterior length of the chest wall in place of the minimum sternovertebral distance.	No validated operative threshold	Pectus carinatum lacks a validated severity index equivalent to those for pectus excavatum; assessment remains largely clinical [1].

Abbreviations: HI, Haller index; CI, correction index; VI, vertebral index; BM/BMX, body–manubrium/body–manubrium–xyphoid index. Definitions and thresholds are drawn from [1,14,19,20,21,22,23]. The Haller index cut-off of 3.25 has not been independently validated, whereas the correction index has [23].

## Data Availability

No new data were created or analyzed in this study. Data sharing is not applicable to this article.
